# LAWS, REGULATIONS, AND POLICY: Consumer Protection Act Sees Uneven Launch

**Published:** 2009-10

**Authors:** Richard Dahl

**Affiliations:** Boston freelance writer **Richard Dahl** has contributed to *EHP* since 1995. He also writes periodically for the Massachusetts Institute of Technology

The Consumer Product Safety Improvement Act (CPSIA), passed by Congress in 2008 in response to widespread recalls of lead‐containing toys manufactured in China, imposed new testing and documentation requirements upon manufacturers of juvenile products and gave the Consumer Product Safety Commission (CPSC) new enforcement powers to ensure compliance. Six months after the new law was scheduled to go into effect, however, it is still the subject of controversy and confusion.

The new law set maximum permissible levels of 600 ppm lead (used to inexpensively increase durability, weight, and paint brightness) and 0.1% of three specified phthalates (plastic softeners) for products intended for children aged 12 years and younger. A more stringent lead level was phased in 14 August 2009, when the permissible level dropped to 300 ppm; in two years it will drop again, to 100 ppm. Manufacturers were to ensure their products do not exceed those levels prior to release into the marketplace.

But the testing requirements met strong opposition from manufacturers, who argued that the timeline and content of the rules imposed an unrealistic burden on small and large producers alike. Days before the requirements were to go into effect, the CPSC issued a one‐year stay of enforcement until 10 February 2010 to provide agency staff time to develop guidance on when and how testing is to be done, and on what materials.

Such guidance has begun to emerge. The CPSC announced in the 7 August 2009 *Federal Register* that wood, natural fibers, and gemstones joined selected metals and alloys as materials that are exempt from testing. In addition, the CPSC clarified the conditions that must be present—such as security against leaching—in order for a lead‐containing component part to be deemed “inaccessible” during “normal and reasonably foreseeable use and abuse of the product by a child.” Inaccessible component parts contained inside a product, which typically are not touched or mouthed by a child, also are on the list of testing exemptions.

Another requirement, which did go into effect August 14, calls for manufacturers and importers to include tracking labels on all children’s products manufactured after that date. Tracking labels must provide information about when and where a product was made, which will help officials locate specific children’s products in the event of a safety recall. The rule also originally required that manufacturers include specific information such as batch and lot numbers, but small manufacturers objected to this provision because they lack the production volume to justify the sophisticated batching systems employed by larger manufacturers. In response, the CPSC allowed small manufacturers to comply solely by maintaining adequate records of the components they use.

Despite the confusion surrounding CPSIA, manufacturing interests and children’s health advocates alike agree it is an essential law, albeit a work in progress. “We supported the legislation from day one, as long as it didn’t put small operators out of business,” says Michael Dwyer, executive director of the Juvenile Products Manufacturers Association. “For bigger companies, it might still be a burden, but they have the resources at their disposal [for testing and tracking]. But when you’ve got mom‐and‐pop companies that have to comply with some of these provisions that are just unrealistic, that is a serious concern to us.”

Some of those provisions have been addressed by the CPSC, but Dwyer thinks there are others. The biggest area of concern remains the expense and logistics of testing, he says. For instance, handheld X‐ray fluorescence (XRF) machines provide an affordable method for determining the presence of lead in products but may not provide sensitive enough measures of lead content to meet CPSC requirements when the stay is lifted next February. In the case of lead testing, Dwyer says, “Our advice to our members is to use XRF testing as a marker. If it’s coming up with lead content, make sure you do your due diligence and if necessary perform the necessary wet chemistry testing to verify compliance with the lead‐level limits.”

Rachel Weintraub, director of product safety and senior counsel at the Consumer Federation of America, is an advocate for lead‐free children’s products and a strong CPSIA, but agrees that the stay on testing was a good idea. “There was confusion,” she says. “The CPSC was not as clear as they could have been.” During a 10 September 2009 hearing by the House Subcommittee on Commerce, Trade, and Consumer Protection, newly appointed CPSC chairwoman Inez Tenenbaum pledged to “continue to solicit feedback from all involved parties, and work to implement commonsense rules that are squarely focused on maximizing product safety and reducing administrative burdens.”

Weintraub points out that CPSIA marks a profound change for the CPSC because that agency had never had pre‐market jurisdiction before. She contends the CPSC needs time to work out regulatory details of the law. “When the whole regulatory structure of an industry changes, there are going to be growing pains,” she says. “But once we see how it’s working, I think it will show that our products have changed for the better.”

In the meantime, public health officials recommend that parents wash their children’s hands several times a day in case they have handled lead‐containing products. They also suggest parents prevent children from putting painted, metal, or plastic items in their mouths unless the parent is certain the item is safe.

## Figures and Tables

**Figure:**
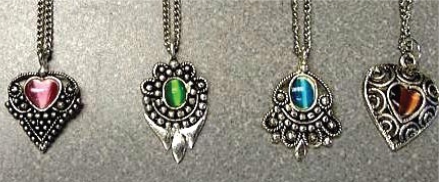
Jewelry may consist of a lead core coated with another material such as nickel. It may also be decorated with lead-containing enamel or assembled with lead solder.

